# Endogenous hepcidin synthesis protects the distal nephron against hemin and hemoglobin mediated necroptosis

**DOI:** 10.1038/s41419-018-0568-z

**Published:** 2018-05-10

**Authors:** Rachel P. L. van Swelm, Madelon Vos, Frank Verhoeven, Frank Thévenod, Dorine W. Swinkels

**Affiliations:** 1 Department of Laboratory Medicine, Radboud university medical center, Radboud Institute for Molecular Life Sciences, Nijmegen, The Netherlands; 2 Institute of Physiology, Pathophysiology & Toxicology, Center for Biomedical Training and Research, University of Witten/Herdecke, Witten, Germany

## Abstract

Hemoglobinuria is associated with kidney injury in various hemolytic pathologies. Currently, there is no treatment available and its pathophysiology is not completely understood. Here we studied the potential detrimental effects of hemoglobin (Hb) exposure to the distal nephron (DN). Involvement of the DN in Hb kidney injury was suggested by the induction of renal hepcidin synthesis (*p* < 0.001) in mice repeatedly injected with intravenous Hb. Moreover, the hepcidin induction was associated with a decline in urinary kidney injury markers 24p3/NGAL and KIM1, suggesting a role for hepcidin in protection against Hb kidney injury. We demonstrated that uptake of Hb in the mouse cortical collecting duct cells (mCCD_cl1_) is mediated by multi-protein ligand receptor 24p3R, as indicated by a significant 90% reduction in Hb uptake (*p* < 0.001) after 24p3R silencing. Moreover, incubation of mCCD_cl1_ cells with Hb or hemin for 4 or 24 h resulted in hepcidin synthesis and increased mRNA expression of markers for oxidative, inflammatory and ER stress, but no cell death as indicated by apoptosis staining. A protective role for cellular hepcidin against Hb-induced injury was demonstrated by aggravation of oxidative, inflammatory and ER stress after 4 h Hb or hemin incubation in hepcidin silenced mCCD_cl1_ cells. Hepcidin silencing potentiated hemin-mediated cell death that could be diminished by co-incubation of Nec-1, suggesting that endogenous hepcidin prevents necroptosis. Combined, these results demonstrate that renal hepcidin synthesis protects the DN against hemin and hemoglobin-mediated injury.

## Introduction

Reactive forms of iron (Fe), such as heme, are increasingly associated with renal injury^1^. Hemolysis and subsequent hemoglobinuria have been related to renal injury in various pathologies including paroxysmal nocturnal hemoglobinuria, favism and sickle cell anemia, but also as potential post-operative complication of cardiopulmonary bypass^2–7^. Also hematuria has been linked with hemoglobin-induced kidney injury, e.g., in patients with IgA nephropathy^4^. At this moment, there are no specific preventive measures or therapies for hemoglobin-induced kidney injury.

Hemolysis leads to cell-free circulating hemoglobin (Hb), which can be filtered by the glomerulus and reabsorbed by the megalin endocytic receptor in proximal tubules (PT)^8,9^. Subsequently, heme is liberated from Hb and exported by the heme exporter FLVCR, used in heme-carrying proteins such as cytochrome P450 enzymes, or converted to bilirubin by heme oxygenase-1 (HO-1). HO-1-mediated catabolism yields intracellular free and reactive Fe^2+^, which is converted to Fe^3+^ by H-ferritin and stored by L-ferritin or exported by ferroportin-1 (FPN1/SLC40A1/IREG1)^10^. The exact mechanisms underlying Hb-induced kidney injury have not been completely elucidated, but appear to be multi-factorial. Oxidative stress plays an important role in tubular damage during hemoglobinuria^11^. Heme redox cycling between ferric and ferryl states generates radical oxygen species that promote tissue damage if their concentration exceeds the catabolic and antioxidant capacity of HO-1^3^. Reactive Fe becomes toxic when Fe storage and export capacities of ferritin and FPN1, respectively, are exceeded^4^. Indeed, increased levels of cellular iron may lead to a regulated form of necrosis named ferroptosis^12^. Ferroptosis has been specifically implicated in acute kidney injury and involves glutathione depletion, oxidative stress and lipid peroxidation^13–15^. Renal tissue of guinea pigs with experimental transfusion-related hemolysis showed increased staining of a lipid peroxide marker for oxidative damage to tissue proteins^16^. Also endoplasmic reticulum (ER) stress and subsequent unfolded protein response (UPR) may be involved. Evaluation of the renal gene transcript response in hemolytic guinea pigs unveiled increased expression of UPR genes, which was confirmed by immunostaining of UPR chaperone HSP70 in tubular epithelial cells. This study also revealed increased response of the ER stress pathway of apoptosis to hemoglobinuria. Furthermore, heme-induced renal apoptosis plays an important role in acute renal failure in rats with glycerol-induced rhabdomyolysis^17^. In addition to ferroptosis and apoptosis, heme toxicity has been associated with another form of regulated cell death, called necroptosis, in macrophages, astrocytes, cortical neurons and endothelial cells^18–21^. Necroptosis is regulated via RIPK3 and can be initiated via several triggers, including inflammatory stimuli and ischemia/reperfusion injury^22^. Indeed, heme can trigger an inflammatory response in patients and experimental animal models for hemoglobinuria, which may be mediated by the toll-like receptor 4 (TLR4)/NF-κB pathway and Interleukin-6 (IL-6)^17,23,24^.

Molecular interactions of intra-tubular Hb have predominantly been described for epithelial cells of the PT. However, since Hb casts have been observed in the distal nephron (DN) after hemolysis^9,25–28^, tubular Hb excess is also likely to affect the DN. Recently, we observed uptake of fluorescently labeled Hb in a mouse cortical collecting duct cell line (mCCD_cl1_), demonstrating the potential of Hb to enter epithelial cells of the DN^29^. In this study, the molecular mechanism of Hb uptake was not elucidated, but it has been shown that proteins are reabsorbed in DN segments via the multi-protein ligand 24p3/NGAL/lipocalin-2 receptor (24p3R; SLC22A17) in case PT are overwhelmed^30,31^. The 24p3 receptor mediates endocytosis of free and Fe-bound 24p3^32–34^, but also facilitates endocytic uptake of other Fe-containing ligands, such as Fe-binding proteins, including transferrin, albumin or methallothionin^30,31^.

Data from multiple clinical observational and experimental studies suggest that the Fe-regulatory hormone hepcidin may protect against heme-mediated kidney injury^29,35–38^. Interestingly, it was the amount of hepcidin present in urine, and not blood, that associated with reduced risk for kidney injury in patients undergoing cardiopulmonary bypass^35,36^. Since all patients had similarly elevated blood hepcidin concentrations^37^, the differentially increased amount of hepcidin in urine could be explained by local renal production. Indeed, hepcidin is synthesized in the kidney, specifically in the DN^29,39,40^.

The present study was conducted to get more insight in the molecular pathways that are involved in renal Hb handling and subsequent injury in the DN and the potential modification of these processes by locally synthesized hepcidin.

## Results

### Repeated Hb administration in mice results in increased renal hepcidin synthesis and adaptation to renal injury

**Fig. 1:**
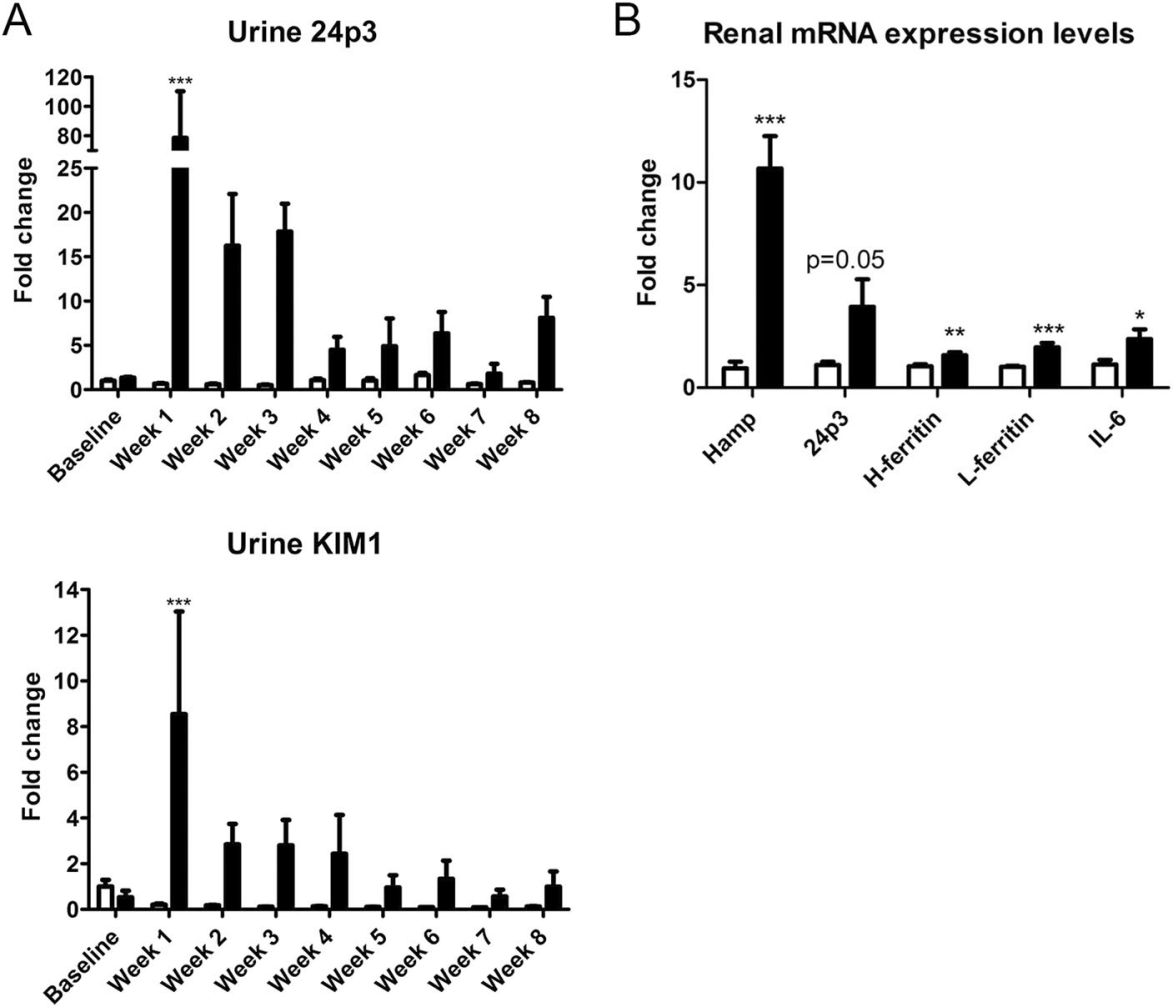
Kidney injury markers in Hb-treated mice. Mice treated with a weekly i.v. injection of saline (control; *n* = 5) or Hb (*n* = 4) for 8 weeks demonstrated increased urinary kidney injury markers 24p3 and KIM1 compared to control (**a**). Increased renal mRNA expression of *Hamp* after 8 weeks of Hb treatment, accompanied by increased *IL-6*, *24p3*, *H-ferritin*, and *L-ferritin* (**b**). Data in panel A was analyzed with Two-way ANOVA with Bonferroni post-hoc test; data in panel B with Student’s *t*-test. * = *p* < 0.05; ** = *p* < 0.01; and *** = *p* < 0.001 compared to control

C57Bl/6 mice were injected with i.v. Hb once weekly for 8 weeks to study the effect on renal hepcidin synthesis in relation to injury. After each Hb injection, urine was collected to assess urinary kidney injury markers 24p3/NGAL and kidney injury molecule 1 (KIM1). Urinary levels of 24p3 and KIM1 were significantly elevated in Hb-treated mice after the first injection (Fig. 1a), indicating early Hb-induced kidney injury. The levels of both injury markers subsequently declined over time, but remained elevated compared to control. Renal mRNA expression levels of *24p3* (*p* = 0.05), *H-ferritin* (*p* < 0.01), *L-ferritin* (*p* < 0.001), and *IL-6* (*p* < 0.05; Fig. 1b), measured at the end of the study were increased. Moreover, renal mRNA expression levels of hepcidin (*Hamp*) were increased 10-fold in Hb-treated mice (*p* < 0.001) compared to control, which, in view of the known localization of renal hepcidin production^29,39,40^, demonstrates involvement of the DN. The presumed protective effects of hepcidin against Hb-induced renal injury^29,35–38^ may even suggest that local hepcidin synthesis prevented Hb-induced kidney injury.

### Hb is taken up via 24p3R in mCCD_cl1_ cells

**Fig. 2:**
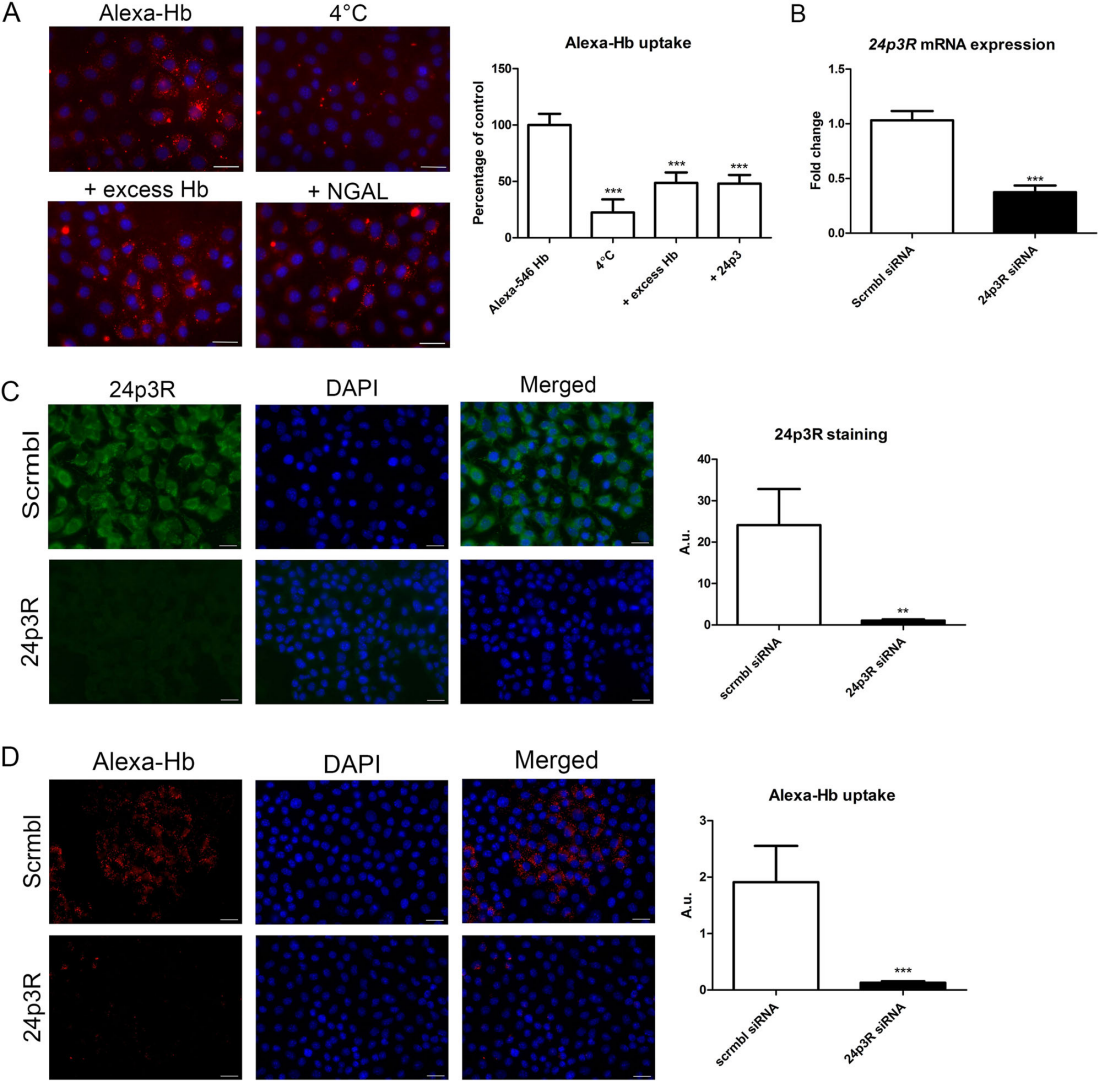
Uptake of Hb by 24p3R in mCCD_cl1_ cells. Uptake of Alexa-546 Hb in mCCD_cl1_ cells after 4 h incubation (**a**) with Alexa-546 Hb alone (Alexa-546 Hb, 1 nM), 4 °C, and with co-incubations of unlabeled excess Hb (+excess Hb, 100 nM) or 24p3 (+24p3, 1 nM). Silencing of 24p3R in mCCD_cl1_ cells resulted in a 60% reduction of mRNA expression level (**b**) and 95% reduction of protein levels as assessed by immunostaining (**c**) compared to scrambled siRNA (scrmbl). MCCD_cl1_ cells treated with 24p3R siRNA demonstrated a 90% reduction in 5 nM Alexa-546 Hb uptake after 1 h (**d**). Panel A: *N* = 2 experiments in duplicate; uptake of Alexa-546 Hb was quantified in 3–4 images per sample; scale bar = 30 µm, *** = *p* < 0.001 compared to Alexa-546 Hb analyzed by One-way ANOVA with Bonferroni’s Multiple Comparison Test. Panel B–D: *N* = 3–4 experiments in duplicate; fluorescence was quantified in 3–4 images per sample; scale bar = 20 µm, ** = *p* < 0.01; *** = *p* < 0.001 compared to scrmbl siRNA, analyzed by Student’s *t*-test

We investigated the role of 24p3R in Hb uptake in mCCD_cl1_ cells by competitive inhibition using fluorescently labeled Hb (Alexa-546 Hb). Non-specific binding of Alexa546-Hb was assessed by incubation at 4 °C. Excess unlabeled Hb (100 nM) was used to determine Hb-specific uptake, and 24p3 (1 nM), the natural high affinity ligand of 24p3R^41^, to study 24p3R-specific uptake. Both significantly reduced uptake of Alexa546-Hb in mCCD_cl1_ cells (both *p* < 0.001; Fig. 2a).

Next, 24p3R was silenced by siRNA to functionally determine its contribution to mCCD_cl1_ Hb uptake. Silencing of 24p3R for 72 h reduced *24p3R* mRNA expression by 60% (*p* < 0.001) and 24p3R protein level by 95% (*p* < 0.01) as assessed by immunofluorescence staining (Fig. 2b-c), compared to scrambled siRNA. Incubation with Alexa-546 Hb for 1 h demonstrated a 90% reduction in Hb uptake in 24p3R silenced mCCD_cl1_ cells (*p* < 0.001; Fig. 2d), indicating that 24p3R is the major route of mCCD_cl1_ Hb uptake.

### Hb and hemin induce hepcidin synthesis and intracellular cell stress in mCCD_cl1_

**Fig. 3:**
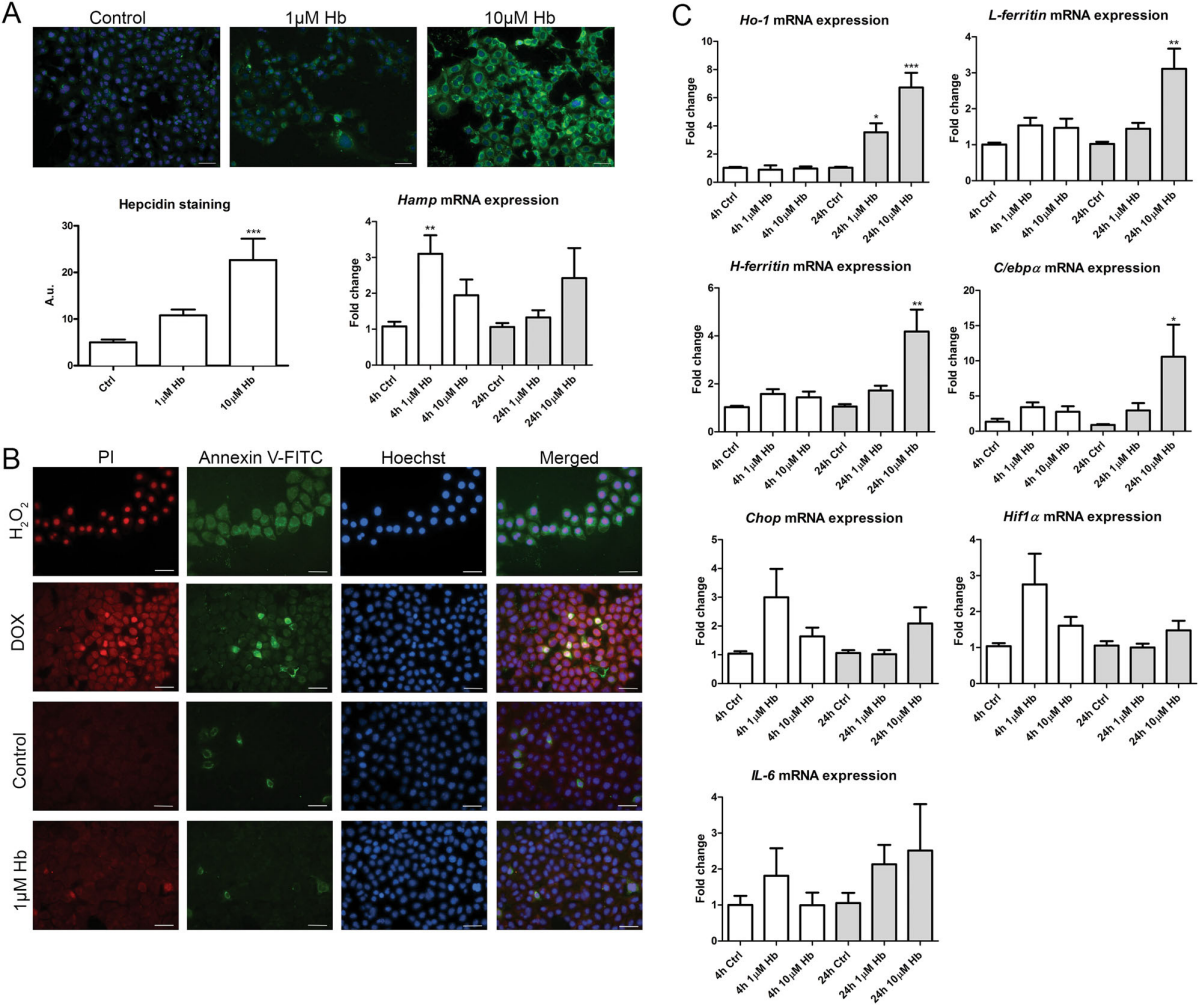
Hb-mediated induction of hepcidin synthesis, cell death and cellular stress. Immunostaining of hepcidin mCCD_cl1_ cells after 24 h hemoglobin (Hb) incubation and *Hamp* mRNA expression levels in mCCD_cl1_ cells after 4 h and 24 h Hb incubation (**a**). Absence of Hb-induced cell death modes necrosis (PI) and apoptosis (Annexin-V FITC) in mCCD_cl1_ cells incubated with Hb for 48 h (**b**). H_2_O_2_ (30 min) served as positive control for necrosis and Doxorubicin (DOX, 24 h) for apoptosis. Incubation with Hb for 4 h (white bars) and 24 h (grey bars) resulted in significant dose-dependent increased mRNA expression of *Ho-1*, *L-ferritin*, *H-ferritin*, and *C/ebpα*, whereas *Chop*, *Hif1α* and *IL-6* were moderately elevated (**c**). *N* = 3 experiments in duplicate; fluorescence was quantified in 3–4 images per sample. Scale bar panel A = 40 µm, scale bar panel B = 30 µm. * = *p* < 0.05; ** = *p* < 0.01; and *** = *p* < 0.001 compared to control (ctrl); analyzed by One-way ANOVA with Bonferroni’s multiple comparison test

Dose-dependent hepcidin synthesis was evident in mCCD_cl1_ cells after 24 h incubation with Hb on protein level (*p* < 0.001 for 10 µM) and after 4 h of Hb incubation on mRNA expression level (*p* < 0.01 for 1 µM; Fig. 3a). Cell death by propidium iodide (PI) and Annexin V-FITC staining was not observed when cells were incubated with Hb for 48 h (Fig. 3b). Despite the absence of cell death, Hb incubations of 4 and 24 h led to cellular stress as indicated by mRNA expression levels of various markers (Fig. 3c). The significant and dose-dependent induction of *Ho-1* mRNA at 24 h (*p* < 0.05 for 1 µM; *p* < 0.001 for 10 µM) revealed Hb catabolism, whereas the induction of *H* and *L-ferritin* mRNA at 24 h (both *p* < 0.01 for 10 µM) suggested increased intracellular Fe handling and storage. Expression of CCAAT/enhancer-binding protein α (C/ebpα) mRNA, a transcription factor reported to induce hepcidin synthesis in hepatocytes^42^, was significantly elevated after 24 h Hb incubation (*p* < 0.05 for 10 µM). Also mRNA expression levels of C/ebp homologous protein (Chop), Hypoxia inducible factor 1α (Hif1α) and *IL-6* were increased, although not significantly, which might hint towards ER stress, oxidative stress and inflammation, respectively^43,44^. Overall, most cell stress markers were induced already after 4 h of Hb incubation, some of which became statistically significant after 24 h (*Ho-1, H-ferritin, L-ferritin, C/ebpα*). Combined these results demonstrated that Hb is catabolized in mCCD_cl1_ cells and induces cellular stress, but does not lead to cell death.

**Fig. 4:**
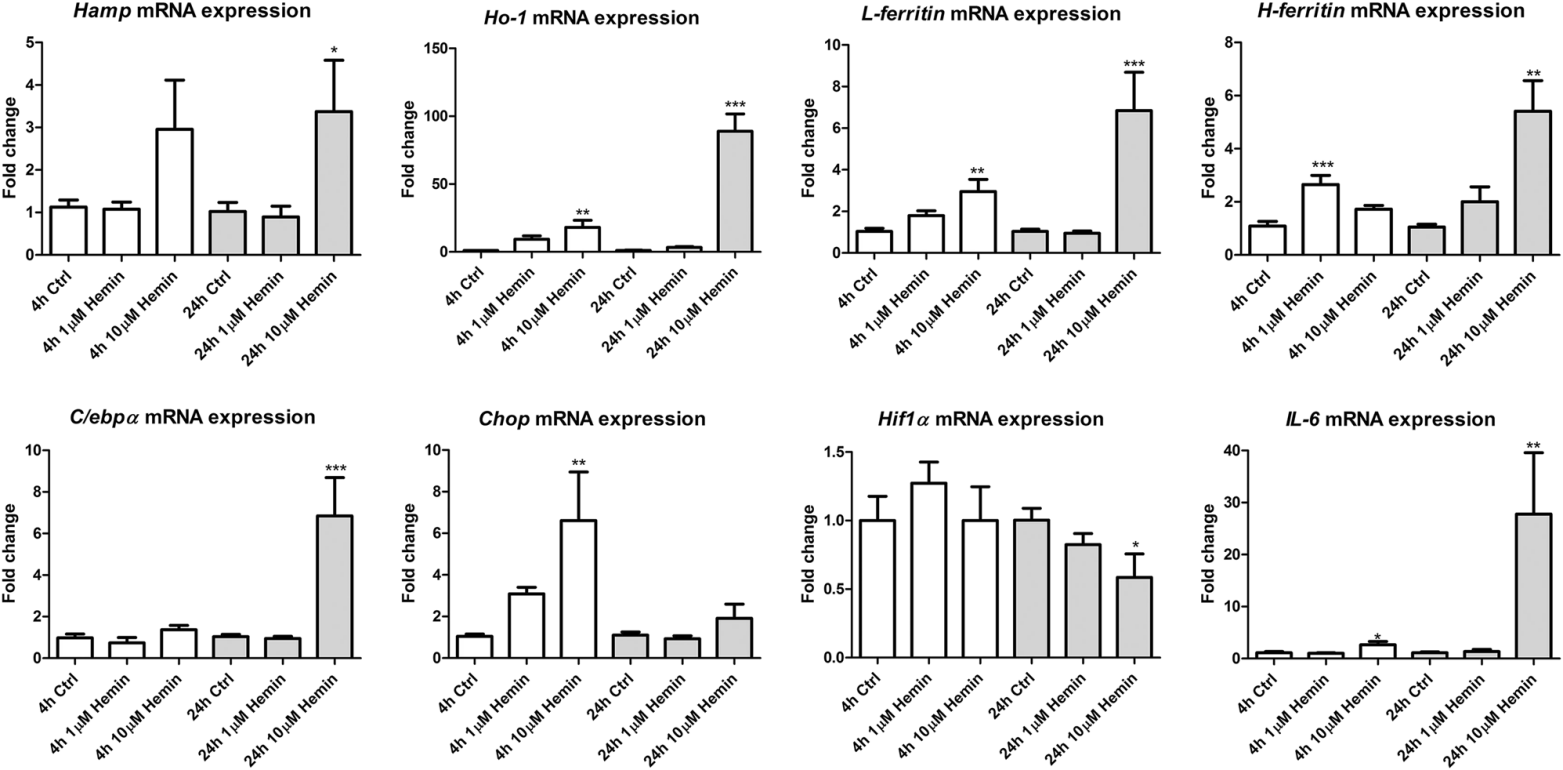
Hemin-induced cellular stress. Incubation of mCCD_cl1_ cells with hemin for 4 h (white bars) or 24 h (grey bars) resulted in significant and dose-dependent increases in mRNA expression of *Hamp*, *Ho-1*, *L-ferritin*, *H-ferritin*, *C/ebpα*, *Chop*, and *IL-6*, whereas mRNA expression of *Hif1α* was significantly reduced compared to control (ctrl). *N* = 3 experiments in duplicate. * = *p* < 0.05; ** = *p* < 0.01; and *** = *p* < 0.001 compared to control; analyzed by One-way ANOVA with Bonferroni’s multiple comparison test

To investigate whether cellular stress is caused by the Fe-containing heme component of Hb, mCCD_cl1_ cells were incubated with 1 or 10 µM hemin for 4 h and 24 h (Fig. 4). *Hamp* mRNA expression was induced after 4 h and 24 h after 10 µM (*p* < 0.05 for 24 h). In concurrence, 10 µM hemin incubation for 24 h resulted in significantly induced mRNA expression levels of *IL-6* (*p* < 0.01*)*, *Ho-1* (*p* < 0.001), *L-ferritin* (*p* < 0.001), *H-ferritin* (*p* < 0.01), and *C/ebpα* (*p* < 0.001), and reduced *Hif1α* mRNA expression (*p* < 0.05). Since hemin is readily taken up by the cell and quickly catabolized by HO-1^45^, we expected to find significant effects on Fe metabolism and cell stress markers already at 4 h after exposure. Indeed, mRNA expression of *Ho-1*, *L-ferritin*, *Chop*, and *IL-6* were all significantly elevated after 4 h of 10 µM hemin incubation, whereas *H-ferritin* was increased by 1 µM hemin.

### Silencing of hepcidin in mCCD_cl1_ cells aggravates cellular stress and induces apoptosis in response to Hb and hemin

**Fig. 5:**
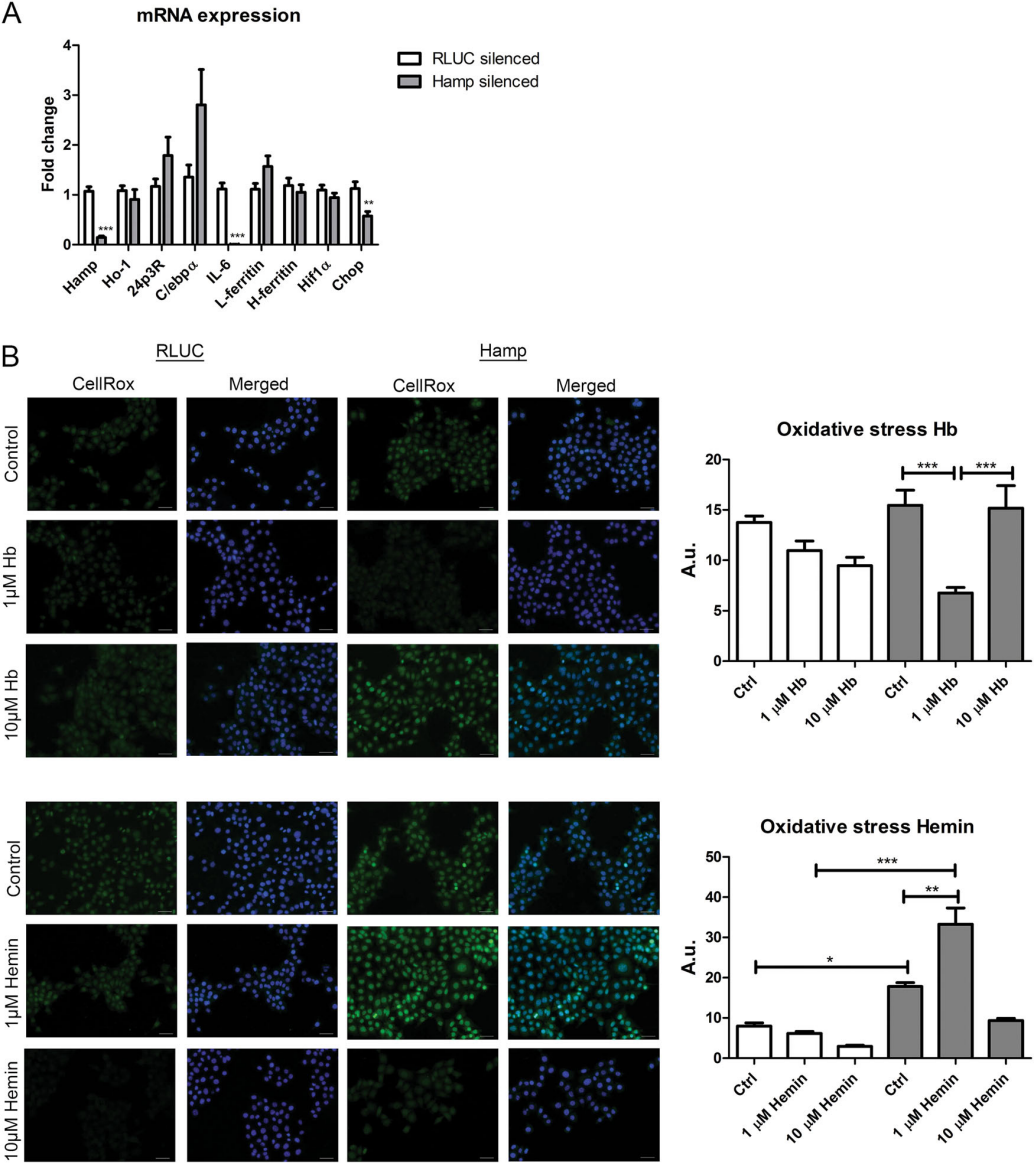
Cellular stress after hepcidin silencing in mCCD_cl1_ cells. MCCD_cl1_ cells treated with Hamp siRNA demonstrate a 90% reduction in *Hamp* mRNA expression level compared to their negative controls treated with RLUC siRNA (**a**). Expression levels of *IL-6* and *Chop* are also significantly reduced. Hamp silenced mCCD_cl1_ cells (grey bars) show increased baseline oxidative stress (**b**) compared to RLUC silenced cells (white bars) and enhanced oxidative stress response after 4 h incubation with Hb (10 µM) or hemin (1 and 10 µM). Panel A: *N* = 6 experiments in duplicate; panel B: *N* = 2 experiments in duplicate. CellRox green fluorescence was quantified in 3–4 images per sample. Scale bar = 40 µm, merged = CellRox + DAPI. * = *p* < 0.05; ** = *p* < 0.01; and *** = *p* < 0.001; data in panel A was analyzed by Student’s *t*-test, data in panel B by One-way ANOVA with Bonferroni’s multiple comparison test

**Fig. 6:**
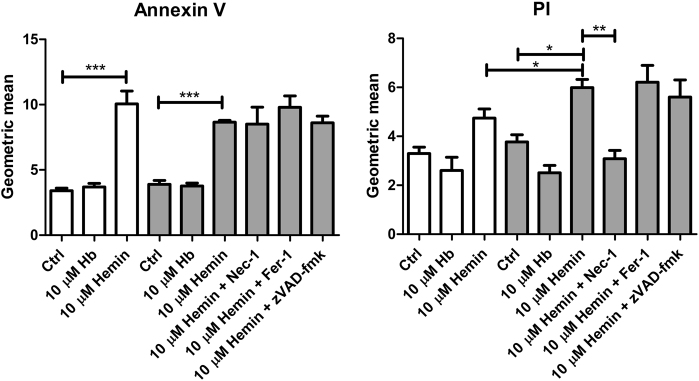
Hemin-induced necroptosis in hepcidin silenced mCCD_cl1_ cells. Incubation with hemin for 4 h resulted in similarly increased Annexin V-FITC signal in both RLUC (white bars) and Hamp silenced (grey bars) mCCD_cl1_ cells, whereas PI was more increased in Hamp silenced mCCD_cl1_ cells. Co-incubation with the necroptosis inhibitor Nec-1, but not with ferroptosis inhibitor Fer-1 or apoptosis inhibitor zVAD-fmk, reduced hemin mediated PI signal in Hamp silenced cells, indicating necroptosis. *N* = 3–4 experiments in duplicate. * = *p* < 0.05; ** = *p* < 0.01; and *** = *p* < 0.001, analyzed by one-way ANOVA with Bonferroni’s multiple comparison test

Hamp siRNA was used to silence hepcidin, with Renilla luciferase (RLUC) siRNA as negative control. *Hamp* mRNA expression levels were lowered by 85% in Hamp siRNA treated cells compared to RLUC controls (*p* < 0.001; Fig. 5a). Surprisingly, Hamp silencing also significantly reduced *IL-6* (*p* < 0.001) and *Chop* (*p* < 0.01) mRNA expression, suggesting that hepcidin exerts a physiological signaling function in mCCD_cl1_ homeostasis. We analyzed the effect of Hamp silencing on Hb and hemin induced oxidative stress by CellRox Green staining after 4 h incubation (Fig. 5b). Interestingly, untreated Hamp silenced mCCD_cl1_ cells had higher oxidative stress levels compared to RLUC controls. Incubation with 1 µM hemin induced significantly more oxidative stress in Hamp silenced cells compared to RLUC controls (*p* < 0.001) and untreated Hamp silenced cells (*p* < 0.01). Surprisingly, oxidative stress was significantly reduced in Hamp silenced cells treated with 1 µM Hb compared to untreated cells (*p* < 0.001). However, since the oxidative stress levels also dose-dependently decreased in both Hb and hemin-treated RLUC controls, in both conditions these reductions may reflect an adaptive response caused by antioxidant mechanisms. Then, cell death was assessed by means of Annexin V and PI FACS analysis in Hamp silenced mCCD_cl1_ cells exposed to 4 h 10 µM Hb and hemin (Fig. 6). Annexin V was similarly significantly induced by hemin exposure, but not Hb exposure, in RLUC and Hamp silenced cells (*p* < 0.001). PI was not increased by Hb, but hemin exposure significantly increased PI in Hamp silenced cells compared to control (*p* < 0.05), but not in RLUC silenced cells. The hemin-mediated induction of PI was significantly reduced to control levels by co-incubation of Nec-1, an inhibitor of necroptosis, whereas co-incubation with an inhibitor for apoptosis (zVAD-fmk) or ferroptosis (Fer-1) had no effect. Moreover, none of the inhibitors had any effect on the hemin-induced Annexin V induction.

**Fig. 7:**
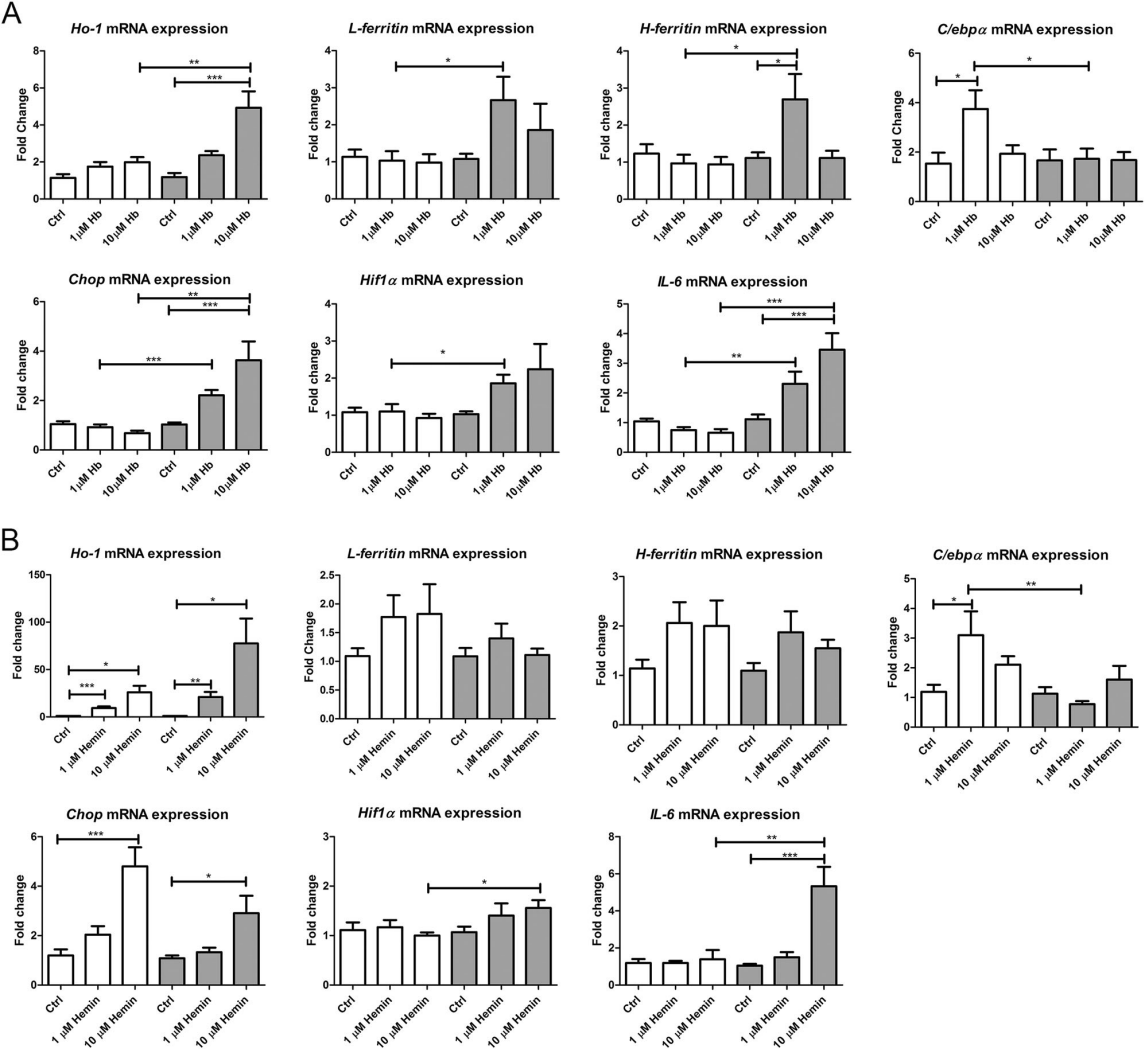
Cellular stress in hepcidin silenced cells incubated with Hb or hemin. MCCD_cl1_ cells treated with Hamp siRNA and incubated with hemoglobin (Hb, **a**) or hemin (**b**) for 4 h demonstrated significant changes in mRNA expression levels of *Ho-1*, *L-ferritin*, *H-ferritin*, *C/ebpα*, *Chop*, *Hif1α*, and *IL-6* compared to cells treated with RLUC siRNA. Changes in mRNA expression levels after Hb or hemin incubated are depicted as fold change relative to their untreated controls in either RLUC or Hamp silenced mCCD_cl1_. *N* = 3 experiments in duplicate. * = *p* < 0.05; ** = *p* < 0.01; and*** = *p* < 0.001, analyzed by one-way ANOVA with Bonferroni’s multiple comparison test

Finally, we analyzed mRNA expression levels of the markers for Fe metabolism and intracellular stress in RLUC and Hamp silenced cells in response to 4 h Hb and hemin incubation (Fig. 7). Hamp silencing resulted in higher *Ho-1* and *IL-6* induction after both Hb and hemin treatment compared to RLUC controls with the same treatment. Increased *Hif1α* mRNA expression levels in Hamp silenced cells in response to Hb and hemin support increased oxidative stress as observed by CellRox green staining. Conversely, the induction of *C/ebpα* in RLUC controls as a result of Hb and hemin exposure was abolished in Hamp silenced mCCD_cl1_ cells, which may be the result of the concurrently increased *Chop* expression, an inhibitor of *C/*ebpα^46^. *L* and *H-ferritin* mRNA expression levels were increased in Hb-treated Hamp silenced cells, but not with hemin incubation. Together, these results demonstrate increased oxidative, inflammatory and ER stress after Hb and hemin exposure in Hamp silenced mCCD_cl1_ cells leading to cell death characterized as necroptosis, with more pronounced effects of hemin compared to Hb.

## Discussion

Hemoglobinuria is associated with kidney injury in many pathologies involving hemolysis. Our knowledge of renal Hb handling and the molecular mechanisms involved in its potential toxicity is incomplete and mostly focused on the PT. Here, we assessed that Hb may also be taken up in the DN through 24p3R and is able to cause cellular stress. Moreover, we demonstrated that local hepcidin synthesis possibly protects against Hb-induced injury.

Our results suggest that hemoglobinuria not only affects the PT, known to facilitate bulk protein reabsorption, but can also reach the DN, since (i) Hb casts have been observed in the DN lumen^9,25–28^, (ii) Hb injections in mice increased renal hepcidin synthesis located in the DN, (iii) Hb can be taken up in the cortical collecting duct cells through 24p3R and, (iv) Hb exposure causes cellular stress in cortical collecting duct cells. The detrimental effects of Hb exposure observed in mCCD_cl1_ cells are likely the result of heme catabolism and subsequent Fe liberation as indicated by the similar, but more pronounced, results obtained after hemin incubation. Although hemin can be readily catabolized by HO-1^45^, which was induced after hemin incubation, we cannot rule out the possibility that hemin affects other pathways. Heme can trigger an inflammatory response by activating TLR4 signaling^47^, as observed in endothelial cells during intravascular hemolysis in a murine model of sickle cell disease^48^. In addition, TLR4 was detected in mouse DN by in situ-hybridization^49^. Nevertheless, it has been demonstrated by Nath et al. that the nephrotoxicity of heme is not solely attributable to TRL4 signaling^50^.

**Fig. 8:**
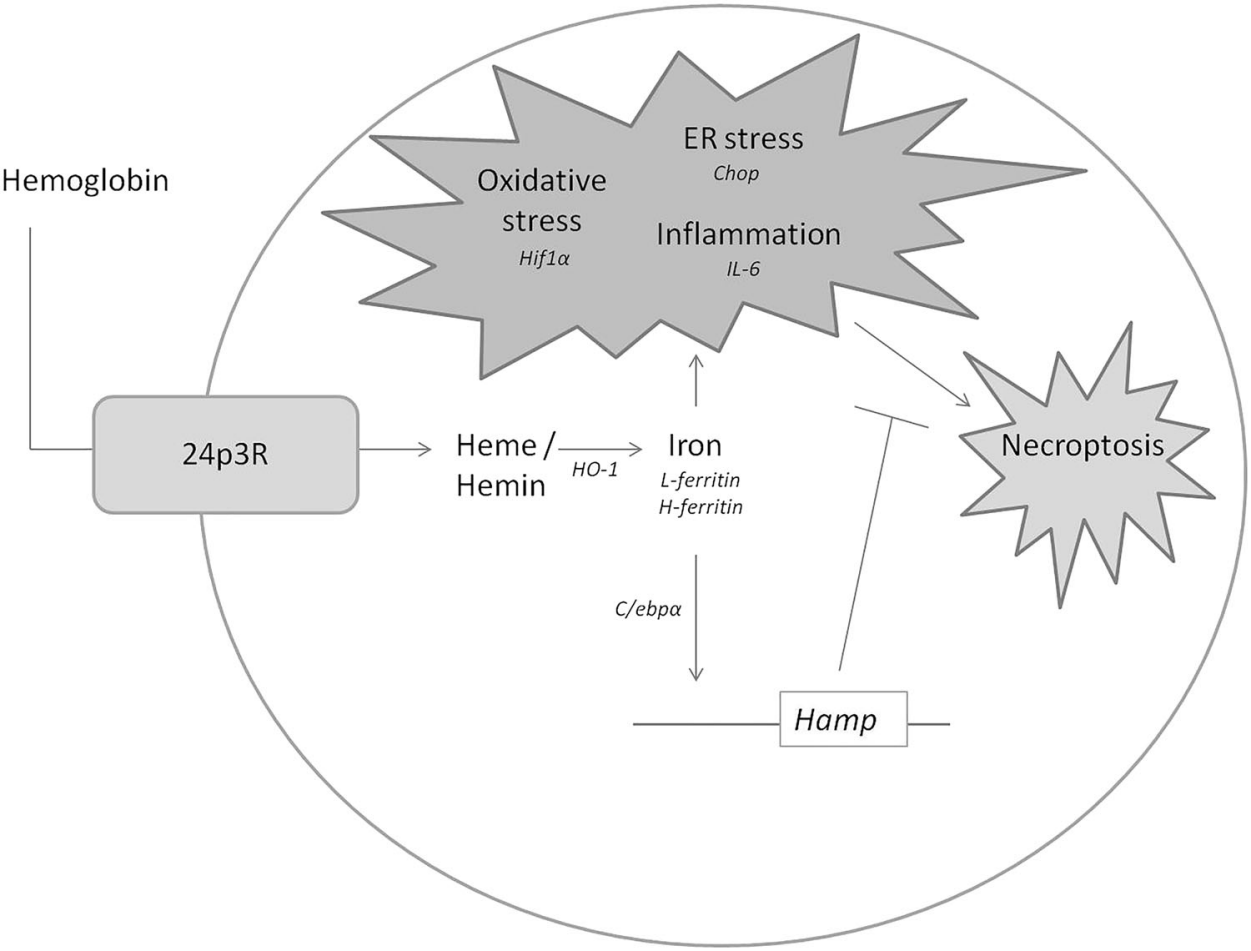
Schematic representation of the proposed mechanisms involved in hepcidin-mediated protection against heme-mediated injury in mCCD_cl1_ cells. The results suggest that Hb is taken up via the 24p3R in mCCD_cl1_ cells, after which the heme-group is liberated and catabolized by HO-1 to yield reactive iron. Initially, reactive iron is metabolized and safely stored (*H-ferritin* and *L-ferritin*), but when intracellular iron levels exceed the capacity for safe storage, excess iron may cause inflammation (*IL-6*), oxidative stress (*Hif1α*) and ER stress (*Chop*). These deleterious pathways can all lead to necroptosis, but simultaneous induction of hepcidin (*Hamp*) synthesis, possibly via *C/ebpα*, prevents cell death. The gene products typed in *Italic* represent the markers measured in the study

The absence of cell death in Hb and hemin treated mCCD_cl1_ cells may be explained by the cellular synthesis of hepcidin, suggested to protect against Hb-mediated kidney injury^29,35–38^. Indeed, siRNA knockdown of hepcidin greatly potentiated the detrimental effects of Hb and hemin in terms of cellular stress and cell death. The proposed mechanisms involved in Hb handling, Hb injury and hepcidin-mediated protection have been summarized in Fig. 8. Interestingly, Hamp silencing alone already reduced *IL-6* and *CHOP* mRNA expression and increased oxidative stress compared to RLUC silenced cells. This may indicate that hepcidin fulfills an important physiological function in mCCD_cl1_ cells and that silencing of hepcidin may consequently result in cell stress, even without an external trigger. Since only Nec-1 was able to inhibit the hemin-induced cell death as indicated by PI in Hamp silenced cells, we postulate that the mechanism of cell death involved in our experiments is necroptosis. Whereas detrimental effects of iron toxicity have been related to ferroptosis^13,14,51^, toxic effects of heme and hemoproteins have been specifically associated with necroptosis^18–21,52^. In our study, the induction of *HO-1*, *L-ferritin* and *H-ferritin* and increased levels of intracellular oxidative stress in hamp silenced cells would suggest increased yield of reactive iron from heme, which could have led to mechanisms of cell death characterized as ferroptosis. Although it has been demonstrated that Nec-1 has anti-ferroptotic effects^22^, the lack of response on PI signal after co-incubation with Fer-1, suggests necroptosis rather than ferroptosis to be involved in our experiments. Nevertheless, it has been reported that both ferroptosis and necroptosis may be involved in a single pathology^14,15,53^. Hb and hemin mediated cell stress involve processes that have been associated with both ferroptosis (oxidative stress^13^, ER stress^54^) and necroptosis (inflammation^22^, ER stress^55^), which might suggest that both forms of regulated cell death may be involved. We have only investigated the effects of hemin induced cell death in Hamp silenced cells after 4 h of hemin exposure. In this time window, only hemin, but not Hb, resulted in cell death. Possibly, the necroptosis observed in this time window is induced by heme directly, either via TLR4, as indicated by *IL-6* upregulation, or ER stress as indicated by *Chop* induction, whereas longer exposure to hemin or Hb would yield excessive amounts of intracellular radical iron that leads to ferroptosis as a result of oxidative stress and lipid peroxidation. Indeed, mixed ferroptosis and necroptosis has been observed in human primary cortical neurons exposed to hemin^56^.

Irrespective of the mechanisms of cell death, our findings agree with previous studies that hepcidin has protective effects on iron-related cell death. Hsieh et al. found that local hepcidin synthesis was needed to abolish Fe^2+-^induced apoptosis in human cardiomyocytes, possibly through regulation of GATA-4 and Bcl-2^57^. Reduced renal tubular apoptosis was also observed in a mouse model of ischemia-reperfusion kidney injury after use of a hepcidin-inducing furin inhibitor^58^. Alternatively, it has been suggested that hepcidin may act as a chelator for reactive Fe^59–61^. Furthermore, systemically administered hepcidin was shown to reduce inflammation in Hb–treated mice^29^ and oxidative stress in murine ischemia/reperfusion kidney injury^38^. Since many of these stress pathways are intertwined it is difficult to determine the exact mechanism(s) involved, which may differ between locally synthesized and circulating hepcidin.

It remains to be elucidated what initiates hepcidin synthesis in response to Hb or heme. We found an upregulation of *C/ebpα*, which could be induced during Hb or heme exposure through IL-6 or TNFa^62,63^, but other mechanisms involved in Hb and heme catabolism could be responsible for hepcidin induction. For instance, oxidative stress can result in hepcidin induction through H_2_O_2_ as demonstrated in hepatocytes^64^. Moreover, heme-induced oxidative stress triggers antioxidant responses via Nrf2, which controls HO-1 expression^65^, but can also trigger hepcidin synthesis, as was shown for phytoestrogens-induced hepcidin activation in hepatocytes^66^. The ER-stress activated transcription factor CREBH can induce hepcidin synthesis by binding to and transactivating the hepcidin promoter in response to toxins or accumulation of unfolded proteins^67^. Finally, inflammation via IL-6 is known to upregulate systemic hepcidin synthesis^68^, and during hemoglobinuria, heme can evoke an IL-6 response through NF-κB that could, by analogy, elicit renal hepcidin synthesis^4,69^.

In conclusion, the results of our study indicate that the DN may play a far more important role in hemoglobinuria than previously assumed in terms of Hb handling and hepcidin synthesis. We advocate studies aiming to unravel the combination and sequence of molecular mechanisms sparked by renal epithelial cells of the proximal and distal tubular segments during hemoglobinuria, which will be essential for a better understanding of the events leading to kidney injury and will define approaches to find preventive or therapeutic measures against Hb-induced renal injury.

## Materials and methods

### Animal studies

All experiments were approved by the local Animal Welfare Committee of the Radboudumc (Nijmegen, the Netherlands; DEC 2012-293) in accordance with the guidelines of the Principles of Laboratory Animal Care (NIH publication 86-23, revised 1985). Male C57Bl/6 N mice (Charles River) of 8–11 weeks of age were housed under controlled conditions with pulverized standard chow and water ad libitum and randomly assigned to a treatment group.

Human Hb (Sigma-Aldrich, the Netherlands) was dissolved in saline (20 mg/mL) and injected via the tail vein, 250 µL/mouse, weekly for 8 weeks. 24 h urine samples were collected at baseline or immediately after each Hb injection. Kidney tissue was collected in liquid nitrogen and stored at −80 °C or in 4% formalin O/N before imbedding in paraffin.

### Enzyme-linked immunosorbent assay (ELISA)

The concentration of 24p3 and KIM1 were determined in urine samples using the DuoSet ELISA development kits from R&D systems (DY1857 and DY1817) according to manufacturer’s protocol.

### Cell culture

The mCCD_cl1_ cell line was established by Rossier et al. and cultured as described. Cells were used for experiments between passage 26 and 34^70^.

Human Hb (Sigma-Aldrich, the Netherlands) was labeled with an Alexa Fluor 546 protein labeling kit (Invitrogen), according to manufacturer’s instructions. Cells were grown on glass cover slides and incubated with 1–5 nM Alexa-546 labeled Hb (Alexa-Hb), unlabeled Hb (100 nM; Sigma-Aldrich) and 24p3 (mouse, 1 nM; Enzo Life Sciences) in serum free medium. Hb was dissolved in PBS and hemin in sterile water (supplemented with final concentrations of 50 mM NaOH and 250 mM Tris base; all Sigma-Aldrich), both used at final concentrations of 1 and 10 µM.

For silencing, cells were transfected using Lipofectamine RNAiMAX (Invitrogen) according to manufacturer’s instructions and siRNA against 24p3R or negative control (scrambled; both 25 nM), and against hepcidin (EMU174481) or negative control against renilla luciferase (RLUC; EHURLUC, both Sigma-Aldrich) at 10 µM. Cells were incubated with siRNA in antibiotic-free medium with 2% FCS for 6 h, after which the medium was refreshed. Cells silenced for 24p3R or scrambled were further analyzed or incubated with Alexa-546 Hb 72 h after starting transfection, whereas for hepcidin or RLUC silencing cells were analyzed or incubated with Hb or hemin 24 h after starting transfection.

To investigate cell death mechanisms, RLUC and Hamp silenced mCCD_cl1_ cells were incubated for 4 h with 10 µM Hb or hemin alone or in combination with 40 µM Necrostatin-1 (Nec-1, N9037, Sigma-Aldrich), 20 µM Ferrostatin (Fer-1, SML0583, Sigma-Aldrich) or 20 µM zVAD-fmk (ALX-260-020-m001; ENZO).

### RNA isolation and quantitative PCR

RNA was isolated with TRIzol (Life Technologies), according to manufacturer’s instructions.

Quantitative PCR was performed with SYBR Green mastermix (2×; Applied Biosystems) and primers are listed in Supplementary Table 1. Fold change values compared to control or baseline were calculated with the 2^^-ΔΔcT^ formula.

### Immunostaining

Cells were washed with PBS and fixed for 30 min with 4% paraformaldehyde, permeabilized with 1% sodium dodecyl sulfate for 15 min and blocked with 1% bovine serum albumin for 1 h at RT. Primary antibody against hepcidin (Abcam ab30760) and the N-terminus of 24p3R^31^ were both diluted 1:100 in blocking solution and incubated overnight at 4 °C. The second antibody (Goat-anti rabbit, Invitrogen A-11008) was incubated for 1 h at RT diluted 1:600. DAPI (300 nM for 5 min) was used to counterstain nuclei. Hepcidin staining was visualized using a Zeiss ApoTome.2 microscope and imaged using Axiovision 4.8.

### Oxidative stress and cell death staining

CellROX Green reagent (Molecular Probes by Life Technologies C10444) was used according to manufacturer’s instructions. Briefly, cells were incubated with CellRox Green reagent for 30 min at 37 °C, fixed, permeabilized and counterstained with DAPI as described above.

Cell death was visualized with Annexin V-FITC and propidium iodide (PI) staining (both from Abcam ab14085). Cells were incubated with both dyes for 5 min in the dark, fixed and counterstained with DAPI or 0.8 µg/mL Hoechst 33342.

### Annexin V and PI flow cytometry

Cells were washed with PBS and harvested using trypsin. Cell pellets were incubated with binding buffer, Annexin V and PI (ab14085, Abcam) for 5 min in the dark, fixed in 4% paraformaldehyde for 10 min and, finally, dissolved in PBS. Annexin V and PI signal were measured on a FACScalibur flow cytometer (BD Bioscience).

### Statistical analysis

Data were presented as mean ± SEM using GraphPad Prism 5.03 software. Statistically significant differences were calculated using Student’s *t*-test or one-way ANOVA with post hoc analysis wherever appropriate. A *p*-value < 0.05 was considered statistically significant.

## Electronic supplementary material

Supplementary Table 1
