# Supplementary material for: Endogenous hepcidin synthesis protects the distal nephron against hemin and hemoglobin mediated necroptosis
Source: Cell Death Dis. 2018 May 10;9(5):550. doi: 10.1038/s41419-018-0568-z (PMC5945780; doi:10.1038/s41419-018-0568-z)
Supplement: Supplementary file 1 — Supplementary Table 1 [file 41419_2018_568_MOESM1_ESM.docx]

**Supplementary file**

Supplementary Table 1. Primers used for quantitative PCR

| **Gene** | **Forward primer (5’-3’)** | **Reversed primer (5’-3’)** |
| --- | --- | --- |
| *β-actin* (housekeeping gene) | GCTATGCTCTCCCTCACGCCA | CTCTTTGATGTCACGCACGAT |
| *HPRT* (housekeeping gene) | GCTGGTGAAAAGGACCTCTCG | CCACAGGACTAGAACACCTGC |
| *Hamp* | TTGCGATACCAATGCAGAAG | GGATGTGGCTCTAGGCTATGTT |
| *24p3* | GCCTCAAGGACGACAACATCA | TTCTCTGTCCCCACCGACCAATGC |
| *24p3R* | GATAGACAGGAAGGCAAGGC | GACGGAGTGAACAGAAAGCA |
| *H-ferritin* | GCGAGGTGGCCGAATCT | CAGCCCGCTCTCCCAGT |
| *L-ferritin* | GAAACTCATCAAGAAGATGGGCA | GCTGGTTGTGGCCCCGC |
| *IL-6* | GAGGATACCACTCCCAACAGACC | AAGTGCATCATCGTTGTTCATACA |
| *Ho-1* | CCTCACTGGCAGGAAATCAT | CCAGAGTGTTCATTCGAGCA |
| *C/ebpα* | CAAGAACAGCAACGAGTACCG | GTCACTGGTCAACTCCAGCAC |
| *Chop* | ATGAAGGAGAAGGAGCAGGAGAA | CTTGGTGCAGGCTGACCATG |
| *Hif1α* | TCAGAGGAAGCGAAAAATGGA | AGTCACCTGGTTGCTGCAATAAT |
